# Comprehensive Investigation of Epoxy Adhesives for Structural Applications in Saudi Arabia: Mechanical Performance, Environmental Impacts, and Analysis on Health

**DOI:** 10.3390/polym16223185

**Published:** 2024-11-16

**Authors:** Ahmed D. Almutairi

**Affiliations:** Department of Civil Engineering, College of Engineering, Qassim University, Buraydah 51452, Saudi Arabia; ah.almutairi@qu.edu.sa; Tel.: +966-16-3020655

**Keywords:** epoxy adhesives, mechanical properties, polymer, occupational safety, environment, Saudi Arabia

## Abstract

Epoxy adhesives possess excellent mechanical properties, durability, and stability in harsh environments, making them suitable for producing engineering materials. This study selects four commercially available epoxy adhesives in Saudi Arabia: Epotec YD 128; Sikadur^®^-52 LP; Sikadur^®^-31 CF; and Sikadur^®^-42 MP Slow. Firstly, a comparison of their storage, application, and service temperatures was made, detailing the hazard identification and prevention measures established in accordance with the Occupational Safety and Health Administration (OSHA) guidelines. Subsequently, test samples of the four adhesives were produced, and tensile, compressive, and shear tests were conducted to compare their fundamental mechanical properties. Finally, a gas analyzer assessed the major harmful gases emitted by these epoxy adhesives 120 min after mixing the epoxy resins and curing agents. The results show that Sika 42 exhibits the highest tensile and compressive strengths among other types of adhesives, reaching 75.7 MPa and 133.8 MPa, respectively. It also has the longest pot life of 48 min at elevated temperatures (40 °C), making it suitable for the climatic conditions in Saudi Arabia. However, as a three-component adhesive, its application is complex and associated with the most identified hazards. Sika 31 presents a tensile modulus of up to 10.4 GPa, at least 3.8 times higher than the other adhesives, making it practical for controlling tensile deformation. Additionally, its ultimate shear strain reaches 10.7%, at least 6.6 times higher than the other samples, highlighting its suitability for constructing ductile bonds. After mixing of epoxy resins with curing agents, the presence of NO_2_ and SO_2_ were detected. However, no harmful gases were detected after 120 min, possibly due to the complete curing of the adhesives.

## 1. Introduction

Epoxy resins are a class of synthetic thermosetting polymers that are widely recognized for their exceptional mechanical properties and versatility in various applications [1]. Defined by their ability to cure through a chemical reaction that typically involves a hardener, epoxy resins exhibit relatively high tensile and compressive strengths, which make them particularly suitable for structural applications in load-bearing scenarios [2,3]. With adequate durability and affordability, epoxy resins have been directly employed in the production of construction materials, such as fiber-reinforced polymer (FRP) composites through the impregnation of carbon or glass fibers [4,5], or polymer concrete through mixing with aggregates [6,7]. Benefiting from the excellent resistance of epoxy resins to corrosion and their durability, these materials can be utilized in marine engineering, often serving as alternatives to steel rebar or other steel structural components. These construction materials have been widely used in buildings [8,9], bridges [10,11], concrete reinforcement [12,13], and other facilities. Their durability, combined with their ability to withstand various stresses, makes epoxy resins an invaluable material in construction and civil engineering, ensuring the longevity and integrity of structural components.

One of the defining characteristics of epoxy resins is their rigidity after mixing with hardeners and curing, which provides a firm and durable bonding material. Their low viscosity facilitates excellent penetration into these imperfections, ensuring strong adhesion to other structural members and promoting efficient load transfer [14]. This property is particularly beneficial in circumstances requiring permanent repairs, as epoxy resins establish a long-lasting and resilient bond. Epoxy adhesives have been practically used to bond wood [3,15], FRP [16], concrete, and steel components [17] for structural reinforcement, preventing the progression of cracking or deformation. Particularly, due to the fact that FRP materials cannot typically be welded, epoxy resins are frequently employed for the bonding of such materials. For instance, in bridge engineering, epoxy adhesives have been employed to bond carbon fiber-reinforced polymer (CFRP) sheets to cracked concrete web plates or columns to enhance their strength [18,19]. Epoxy adhesives have also been used to bond CFRP sheets and steel structures for mechanical strengthening. This has become a conventional solution, which has been well-reviewed in the literature [20], where the impact of key characteristics and environmental factors have been evaluated. Epoxy resins are also utilized in the fabrication of pure GFRP flooring systems, where mechanical robustness is evaluated through systematic laboratory mechanical testing on GFRP sandwich structures featuring GFRP plate face-sheets and GFRP tube webs [21,22]. However, concerns have been widely raised regarding the performance of epoxy resins at elevated temperatures. Although epoxy resins are thermosetting materials, their glass transition temperature is only approximately 100 °C, above which their elastic modulus significantly decreases [23,24]. Consequently, extensive temperature testing has been conducted on profiles containing epoxy resins, including epoxy adhesives and FRP materials, to assess their mechanical performance and the effectiveness of temperature mitigation strategies at elevated temperatures.

Epoxy resin has also been used in Portland cement concrete to enhance its mechanical properties, chemical resistance, and durability. The epoxy resin fills the voids in the microstructure between coarse aggregates and solidifies by forming a network, which contributes to improved strength, and reduced porosity, water absorption, and chloride ion penetration [25]. According to experimental results in the existing literature, the addition of epoxy resin to concrete compositions significantly improves the compressive, tensile, and flexural strength of the material [26]. Concrete specimens with epoxy resin also show superior stability and resistance to acid compared to conventional concrete, indicating higher aggregate–epoxy bonding and highlighting the excellent chemical and solvent-resistant properties of epoxy resin [27,28]. The epoxy resin-based concrete demonstrated a higher resistance to aggressive chloride ion penetration compared to conventional concrete, as indicated by a sharp reduction in chloride ion penetration length [29].

Since the last century, epoxy asphalt has been developed to accommodate road paving on steel bridge decks, as traditional asphalt fails to meet the requirements for high traffic volumes and the constraints of pavement structure thickness and weight [30]. Epoxy asphalt is a two-component reactive chemical system used primarily in the construction of high-performance pavements. It consists of epoxy resins and a mixture of epoxy curing agents, base asphalt, and their compatibilizers [31]. This material has its origins in the need for a durable and high-performing binder for road construction, particularly in areas subjected to heavy traffic and extreme weather conditions. The combination of epoxy resins with asphalt creates a binder that is not only chemically reactive, but also exhibits superior mechanical properties, thermal stability, aging resistance, and fatigue resistance. Extensive research has been conducted to understand the rheological properties and the formation of microstructure networks of epoxy asphalt binders. For instance, studies have focused on improving the low-temperature performance of epoxy asphalt binders [32], investigating the microstructures and mechanical properties of a modified epoxy asphalt binder [33,34], and characterizing the properties of epoxy asphalt binders using differential scanning calorimetry [35].

Due to the high fluidity required for filling voids, several types of epoxy resins with low viscosity have also been used for grouting [36,37] and sealing [38], preventing the intrusion of water and moisture into civil engineering structures and other precision instruments. The effects of moisture, water immersion, sea water, and temperature on the duration of epoxy resins and FRP materials have been well investigated in the literature [39,40,41].

However, epoxy resins, along with their curing agents, (i.e., hardeners) and the polymerization process induced by their interaction, may release potentially harmful byproducts. For instance, many epoxy resins contain Bisphenol A (BPA), an endocrine disruptor that poses health risks upon exposure [42]. The curing agents can encompass aliphatic amines, aromatic amines, anhydrides, and polyamides, each of which may contribute to skin sensitization, respiratory irritation, and other health hazards [43]. Modern applications of epoxy adhesives include a diverse array of formulations designed to achieve specific performance characteristics. As a result, these adhesives may incorporate complex chemical components, the ramifications of which, along with their byproducts, remain poorly understood in terms of their impact on health and the environment. There is an urgent need for comprehensive assessments, yet relevant research is significantly limited.

In recent decades, Saudi Arabia has launched ambitious urban development and infrastructure modernization initiatives, encompassing a substantial number of civil engineering projects. Saudi Arabia’s Vision 2030 is a comprehensive economic and social reform plan put forward by Crown Prince Mohammed bin Salman that aims to diversify the country’s economy, reduce its dependence on oil, and improve various sectors such as education, health, tourism, and entertainment [44]. To protect the environment and realize Saudi Arabia’s Vision 2030 by reducing carbon emissions, there is a critical need to develop polymer-based construction materials, including epoxy resins. However, there is a lack of useful data and understanding of their properties, especially for the types available in the Saudi Arabian market. Therefore, the main objective of this project is to examine the mechanical properties and potential hazards on health and environment of several types of epoxies available in the Saudi market.

This study examines four commercially available epoxy adhesives in the Saudi market, namely Epotec YD 128 X supplied from Aditya Birla Chemicals Ltd. in Bangkok, Thailand, Sikadur^®^-52 LP supplied from Sika Limited Watchmead Welwyn Garden City Hertfordshire, United Kingdom, Sikadur^®^-31 CF supplied from Sika (Auckland, New Zealand) Ltd Avondale Auckland, New Zealand, and Sikadur^®^-42 MP Slow supplied from Sika Corporation Lyndhurst (NJ, USA) assessing them in terms of their applicability, occupational health, environmental impact, and mechanical properties. Initially, based on manufacturers’ data, this study analyzes the storage and applicable temperatures of these epoxy adhesives. Subsequently, the hazards associated with their components concerning occupational health and identified preventive measures are evaluated. Then, samples are prepared for tensile, compression, and shear tests to determine and compare the mechanical properties of the different epoxy adhesives. Finally, a flue gas analyzer is employed to monitor the emissions of harmful gases such as CO, NO, and NO_2_ in the first 120 min after mixing with curing agents, to assess their impact on occupational health and the environment.

## 2. Applicable Scenarios and Occupational Safety Information

### 2.1. Product Purposes and Features

Epotec^®^ YD 128 X is a modified, medium-viscosity epoxy resin based on Bisphenol A that exhibits excellent chemical resistance, prevents crystallization during storage, and enhances the wetting of pigments and fillers. This liquid epoxy resin is standard in the industry and can be cured with a broad range of curing agents under ambient conditions or at elevated temperatures. It is usually used in electrical, electronic, and flooring applications.

Sikadur^®^-31 CF (referred to as Sika 31) is a two-part, high-performance, structural epoxy resin designed for a variety of bonding and repair applications in construction. It has high strength, durability, and a resistance to a range of chemicals and environmental conditions, making it ideal for use on concrete, masonry, metal, and other substrates. With its low-viscosity formulation, Sika 31 ensures easy application and penetration into surfaces.

Sikadur^®^-42 MP Slow (referred to as Sika 42) is a three-part, high-performance epoxy resin specifically engineered for structural bonding and repair applications. Its superior adhesive property is similar to Sika 31. The extended working time afforded by the slow curing mechanism allows for greater adaptability in complex applications, facilitating meticulous alignment and application processes.

Sikadur^®^-52 LP (referred to as Sika 52) is a two-part, solvent-free, low-viscosity injection liquid, based on high-strength epoxy resins. It is suitable for use in hot and tropical climatic conditions.

### 2.2. Applicable Temperature

Commercial epoxy adhesives typically have specified temperature conditions for their proper storage, application, and service, as illustrated in Table 1, according to the manufacturers’ data. Epoxy adhesives generally comprise an epoxy resin and curing agents. The primary component of the epoxy resin is usually Bisphenol A (BPA), which remains stable at room temperature; however, it may decompose at elevated temperatures over 150 °C, thus releasing harmful substances. Notably, Bisphenol A can leach from plastics, and therefore epoxy resins are stored in metal containers. Curing agents vary widely and can use aliphatic amines, aromatic amines, anhydrides, and polyamides. These agents can absorb moisture in high-humidity environments, leading to a reduction in concentration and affecting the effectiveness of curing. Elevated temperatures may also cause the degradation or reactions of certain amines, thereby impacting their performance and stability. Consequently, there are specific requirements regarding their storage temperature and humidity. As shown in Table 1, the storage temperatures for Sika 31, Sika 42, and Sika 52 are all between 5 and 30 °C. Epotec, on the other hand, consists solely of epoxy resin and does not include a curing agent; therefore, no specific storage temperature is stipulated. It is specified that the resin can be restored to its original condition by warming it to 55–60 °C if it becomes hazy or crystallizes due to low-temperature storage.

**Figure 1:**
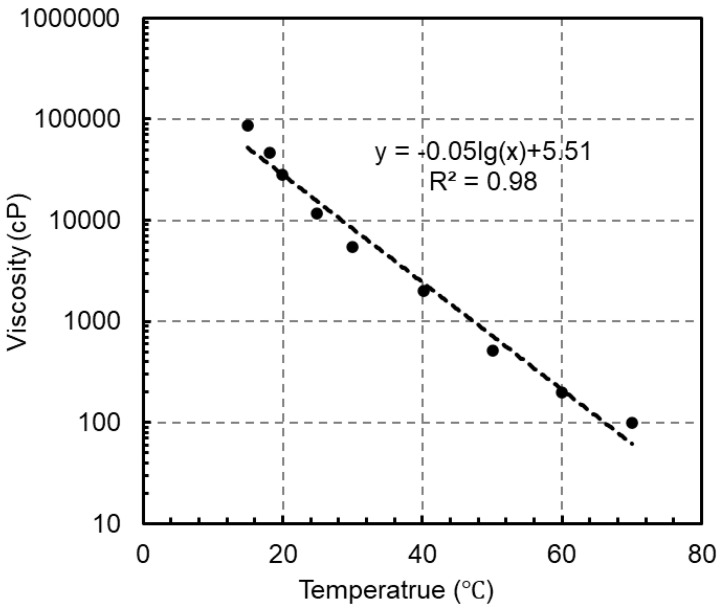
Effects of temperature on the viscosity of Epotec epoxy resin.

When applying epoxy adhesives, the epoxy resin is blended with the curing agents and applied to the substrates. The epoxide groups will react with amine or anhydride groups, leading to a gradual hardening process. The suitable application temperatures, as indicated in Table 1, vary among products; for example, Sika 31 is recommended for use between 10 and 30 °C. However, for specific engineering projects, the appropriate application temperature must be determined by considering both the mixture viscosity and the pot life. Firstly, the workability of the mixture requires a certain level of viscosity. Lower viscosity allows for self-compaction, enabling the adhesive to fill any gaps in the substrate surface. Conversely, excessively high viscosity hinders the epoxy adhesives from achieving sufficient thickness. The viscosity of the epoxy resin and curing agent is significantly influenced by temperature. For instance, Figure 1 illustrates the effects of temperature on the viscosity of the Epotec epoxy resin, indicating a viscosity of 28,510 cP at 20 °C and a drastically reduced viscosity of 1980 cP at 40 °C. As the temperature increases, the viscosity significantly decreases, exhibiting an approximately negative linear relationship with the common logarithm of the application temperature. As viscosity increases, the flowability of epoxy resins decreases, which complicates their mixing with curing agents. A mixture with low flowability is also less effective at filling gaps, potentially resulting in voids and imperfections in the adhesive and inferior bonding performance. Conversely, if the flowability is excessively low, the adhesives may be unable to form their required shapes or achieve sufficient thickness. Therefore, maintaining an appropriate temperature range during the application of epoxy adhesives is essential to ensure adequate workability.

Secondly, after the epoxy resin and curing agent are mixed for a period, they will begin to harden or cure, with the reaction rate being significantly influenced by temperature. The pot life listed in Table 1 refers to the duration during which the mixtures remain workable or usable after blending. For instance, for Sika 52, the pot life at 23 °C is 70 min, while at 40 °C, it is only 10 min. Therefore, the proper pot life should be determined by considering the construction speed and is a critical factor to consider when determining the appropriate application temperature. Saudi Arabia primarily has a desert and semi-arid climate. Most regions experience very little annual rainfall and hot weather, and such temperature and humidity conditions accelerate the reaction between epoxy resins and curing agents, resulting in a shortened pot life. For instance, outdoor temperatures exceeding 40 °C during the summer in Saudi Arabia are not uncommon; under these conditions, Sika 52 has a pot life of only 10 min (see Table 1), which is generally insufficient for construction applications. In this regard, Sika 42 is considered to be more practical.

The cross-linked polymer network is the primary product of the reaction between the epoxy resin and curing agents, and its stability determines the service performance of epoxy adhesives. Due to their high thermal stability, these adhesives are less susceptible to temperature fluctuations. Consequently, cured epoxy adhesives typically maintain stability over extended periods, even in harsh operating environments. Manufacturers’ data sheets of Epotec, Sika 31, and Sika 52 do not specify service temperatures, whereas Sika 42 specifies a service temperature range of −40 to 60 °C, which is adequate for the majority of outdoor environments.

### 2.3. Hazards Identification and Prevention

Bisphenol A (BPA) is classified as an endocrine disruptor, which presents various health risks upon exposure [42]. Research has shown that BPA can accumulate in the bodies of workers in relevant industries. A significant body of experimental evidence illustrates that BPA disrupts the normal functioning of the human reproductive, nervous, and immune systems, as well as adversely affecting embryonic development. Additionally, BPA is linked to the onset and progression of multiple cancer types, including breast and prostate cancers, alongside reproductive tumors in children [45,46]. The curing agents for BPA may include aliphatic amines, aromatic amines, anhydrides, and polyamides, each of which can lead to skin sensitization, respiratory irritation, and other health-related issues [43]. To enhance performance or introduce specific characteristics, a variety of modifiers are incorporated into different epoxy adhesive products. For instance, in Sika 42 B, more than 13 chemical constituents exceed a weight ratio of 1%. Consequently, it is challenging to comprehensively assess the potential adverse health effects associated with epoxy resins, curing agents, and their reaction byproducts. As a precautionary measure, it is typically mandated for workers to wear protective equipment when using epoxy adhesives.

The hazard identification and prevention measures as outlined by the manufacturers in accordance with the Occupational Safety and Health Administration (OSHA) requirements are presented in Table 2 according to the manufacturers’ data sheets. In compliance with these regulations, each part of the epoxy adhesive has a distinct OSHA data sheet. The specifications for the hazard identification codes and the hazard prevention codes are detailed in Table 3 and Table 4, respectively.

Most adhesive parts listed in Table 2 have a special odor, indicating their volatile nature and the potential for inhalation through respiration. These adhesives may cause respiratory irritation (H335), lung damage (H372), and even cancer (H350). Therefore, it is essential not to touch and avoid (P260 and P261) breathing the adhesive parts. As also indicated in Table 2, most adhesive parts are liquid, which increases the likelihood of skin contact and the contamination of clothing, leading to skin irritation or allergic reactions (H315 and H317). Furthermore, the eyes and gastrointestinal tract are generally more sensitive than the skin; hence, these adhesive parts are likely to cause more significant adverse effects on the eyes (H318 and H319) and the gastrointestinal tract (H302), with the potential for fatal outcomes (H304). Consequently, the use of epoxy adhesives necessitates mandatory pre-employment training (P201 and P202). During application, it is essential to wear protective gloves, eye protection, and facial protection (P280).

Due to the potential harm that these adhesive components may cause to aquatic life (H400, H411, or H412 for Epotec, Sika 31, and Sika 52), it is imperative to avoid releasing these adhesives into the environment and to collect any spills (P391). Moreover, it is important to note that measure P501 mandates compliance with local laws and regulations when disposing of hazardous contents or containers. This necessitates the establishment of comprehensive regulations governing the use of epoxy adhesives at the local level to mitigate any adverse impacts on the local residents or the ecological environment.

## 3. Mechanical Property Tests

### 3.1. Tensile Tests

The samples of the epoxy adhesive for the tensile tests were prepared and tested, as per ASTM D638-14 [47]. For Sika 31 samples, the A and B components with a weight ratio of 2:1 were mixed for 5 min with a mixing spindle attached to a slow speed electric drill (max. 300 rpm) until the material became smooth in consistency and had a uniform color. The whole mixture was then poured into a clean container and stirred again for 1 min at a low speed to keep air entrapment at a minimum. After that, the mixture was poured in the mold, and the surface was smoothed with a spatula. The samples were prepared and cured for at least seven days in the laboratory with a constant temperature of about 25 °C. The other epoxy resins and curing agents were blended similarly.

**Figure 2:**
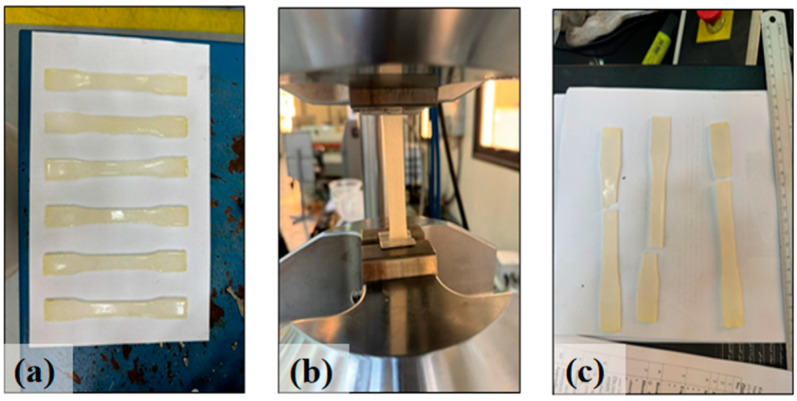
Sika 52 specimens (**a**) before loading, (**b**) under loading, and (**c**) after loading.

The bone-shape sample specimens had a total length of 165 mm and an overall width of 19 mm as shown in Figure 2a. The narrowed middle section had a length of 57 mm and a width of 13 mm. Six repeated specimens were prepared for each type of adhesive, leading to a total of 24 specimens. All specimens were cured for at least 14 days before testing. The tensile loads were applied by an MTS universal testing machine (UTM) as shown in Figure 2b. In the loading process, the total loads and displacements were continuously recorded by the UTM, and the stress and strain of the samples were calculated accordingly. All specimens presented a sudden brittle failure at the ultimate tensile stress, and the failed specimens are shown in Figure 2c.

**Figure 3:**
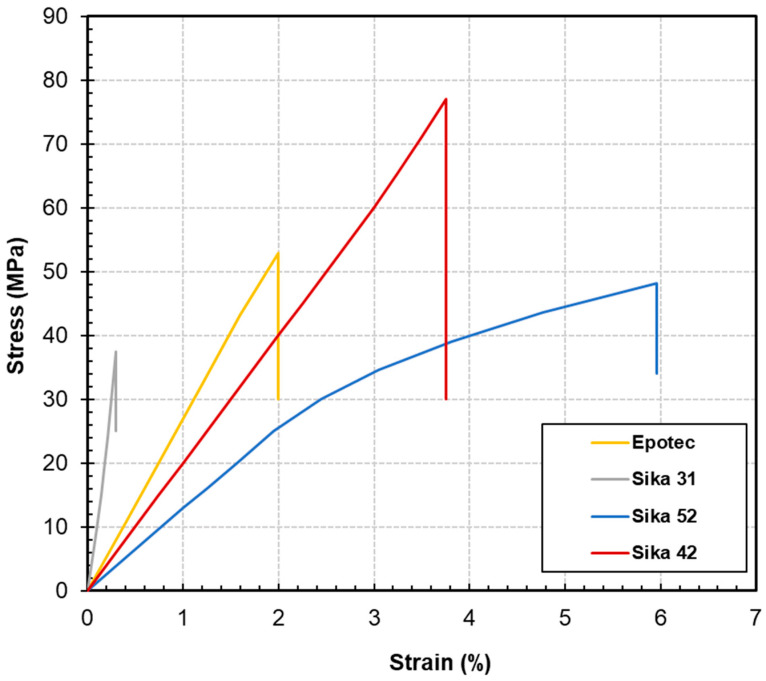
Stress-strain behavior of epoxy adhesive specimens under tension.

The typical stress-strain behavior of the epoxy adhesive specimens under tension are presented in Figure 3 and the tensile properties based on average results among repeated specimens are listed in Table 4. The data presented in Figure 3 indicate that the four types of epoxy adhesives exhibit markedly different stress-strain relationships under tensile loading. Notably, Epotec, Sika 31, and Sika 42 display significant linearity in their responses. According to the data in Table 2, the tensile elastic modulus of Sika 31 is considerably higher than that of the other adhesives, reaching up to 10.4 GPa, making it suitable for applications requiring high stiffness; however, its maximum strain is limited to 0.3%. The modulus values of the other adhesives are all below 2.7 GPa. Additionally, Sika 42 demonstrates a tensile strength that significantly surpasses that of the other adhesives, achieving a value of 75.7 MPa.

In contrast to the linear stress-strain relationships observed in other adhesives, Sika 52 exhibits a bilinear stress-strain relationship, characterized by different slopes prior to reaching a strain of approximately 2.5%. There are several possible reasons for the nonlinear stress-strain curve observed in epoxy adhesives. For example, some epoxy adhesives can exhibit creep (deformation under constant load) and stress relaxation (reduction of stress under constant strain) over time. Additionally, some epoxy adhesives may display viscoelastic properties, meaning that their deformation characteristics depend on both the rate of applied stress and the duration of the load. Compared to the samples of other adhesives, Sika 52 achieves a maximum strain of 5.9%, demonstrating superior ductility. It is worth noting that some researchers have utilized the bilinear stress-strain behavior of Sika 52 to design specialized fiber-reinforced polymer (FRP) structures [48]. This design enables significant deformation under excessive stress, thus facilitating pseudo-ductility as a pre-failure warning mechanism, addressing the drawback of brittle failure typically associated with FRP structures.

### 3.2. Compressive Tests

**Figure 4:**
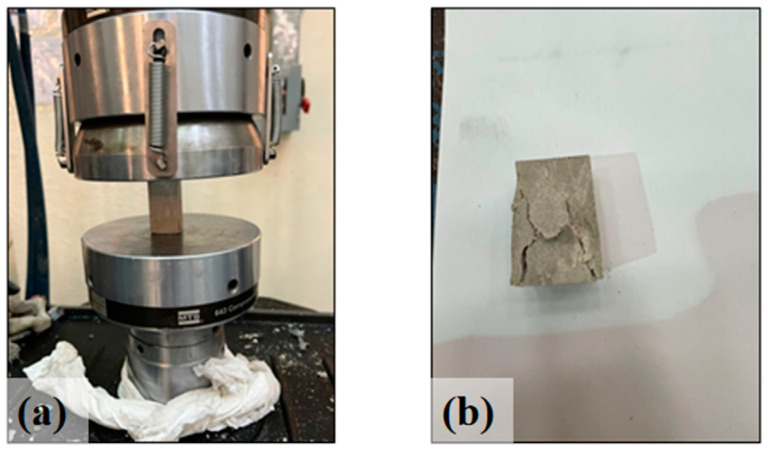
Sika 31 Specimens (**a**) under compression and (**b**) after compression.

The samples of the epoxy adhesive for the compression tests were prepared and tested as per ASTM D695-15 [49]. The dimensions of the rectangular prism compressive specimens were 25 mm × 25 mm × 50 mm (length × width × height). Six replicates were prepared for each type of adhesive, resulting in a total of 24 specimens. All specimens were subjected to a curing period of no less than 14 days prior to testing. Compression loads were applied using an MTS universal testing machine (UTM), as illustrated in Figure 4a. All tested specimens exhibited compression failure as presented in Figure 4b. In the loading process, the total loads and displacements were continuously recorded by the UTM, and the stress and strain of the samples were calculated accordingly.

**Figure 5:**
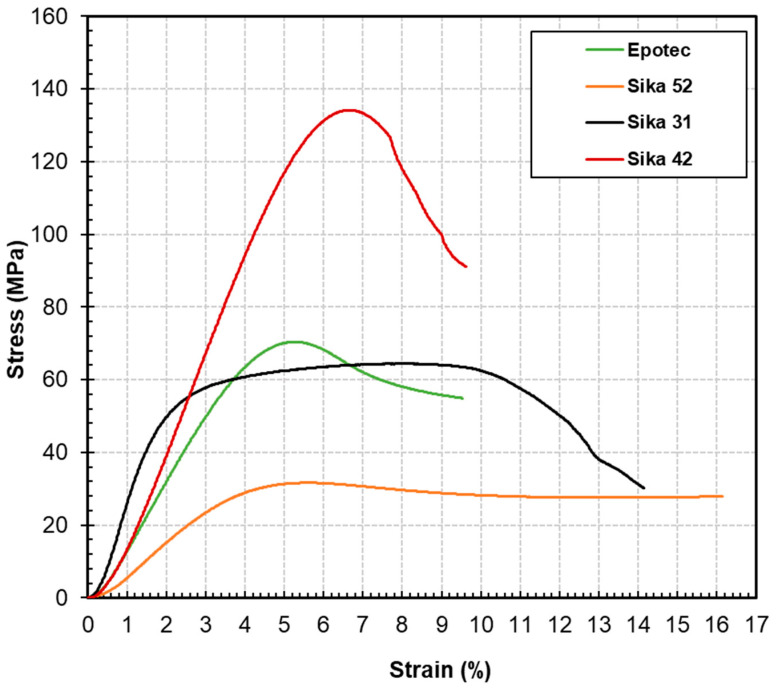
Stress-strain behavior of epoxy adhesive specimens under compression.

The typical stress-strain behavior of the epoxy adhesive specimens under compression is illustrated in Figure 5, and the compression properties, based on the average results of repeated specimens, are presented in Table 5. In contrast to the tensile specimens, the stress-strain relationship exhibited by the compressive specimens demonstrates obvious nonlinear behavior. During the initial phase of loading, strain increases in a nearly linear manner with rising stress; however, after reaching or approaching the maximum stress value, the strain continues to grow towards its maximum without a significant increase in stress.

Comparing the data from the tensile tests in Table 4, the adhesives display a maximum compressive strain that is substantially higher than that of the tensile specimens, reaching at least 9.4% and peaking at 16.1%. This level of ductility is beneficial for structural safety, as it permits substantial deformation prior to failure when the structure nears its compressive load limit, serving as a pre-failure warning. Among the various adhesives tested, Sika 31 exhibits the highest compressive modulus, reaching 2.7 GPa. Sika 42 exhibits a higher compressive strength than the other adhesives, reaching 133.8 MPa. This finding is consistent with the conclusions drawn from the tensile strength evaluations, suggesting that Sika 31 is appropriate for applications requiring deformation control, while Sika 42 is more appropriate for high-stress scenarios. With the exception of Sika 52, the compressive strengths of the adhesives generally exceed their respective tensile strengths (see Table 5 and Table 6).

### 3.3. Shear Tests

**Figure 6:**
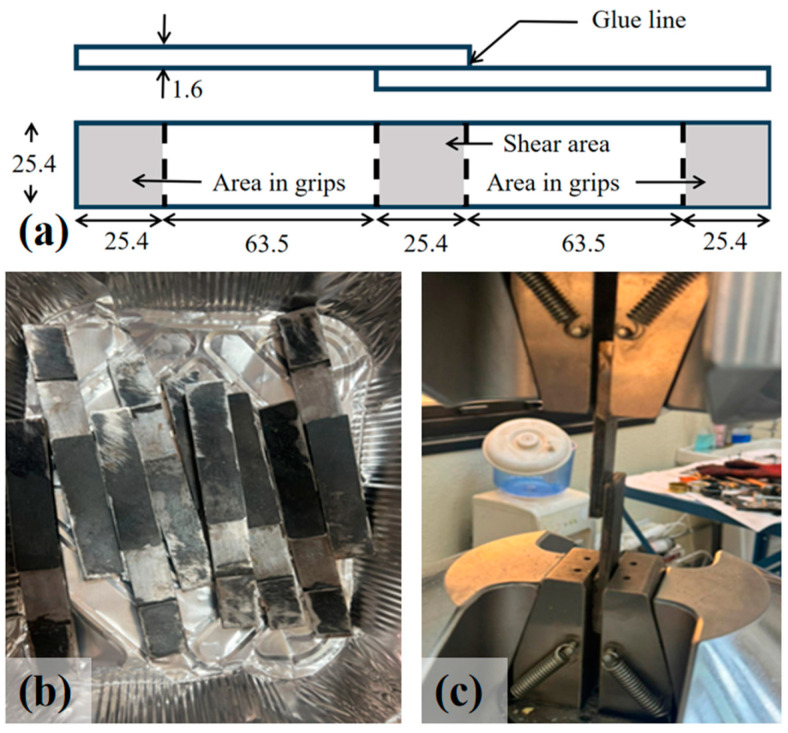
Specimens for shearing tests: (**a**) dimension, unit: mm, (**b**) before tests, and (**c**) test setup.

The samples of the epoxy adhesive for the shear tests were prepared and tested, as per ASTM D1002-10 [50]. In the shear tests, steel plates with a length of 57 mm and a width of 13 mm were first prepared as shown in Figure 6. The steel surfaces were prepared thoroughly by blast cleaning and vacuuming to avoid contamination and to ensure the correct dew point conditions. Every two plates were bonded by the epoxy adhesives with a bonding length of 25.4 mm. The adhesive layer had a thickness of 1 mm. The 25.4 mm section at each end was for clamping. Six repeated specimens were prepared for each type of adhesive, leading to a total of 24 specimens, as shown in Figure 6b. All specimens were cured for at least 14 days before testing. The tensile loads were applied by an MTS universal testing machine (UTM) as shown in Figure 6c. In the loading process, the total loads and displacements were continuously recorded by the UTM, and the stress and strain of the samples were calculated accordingly.

**Figure 7:**
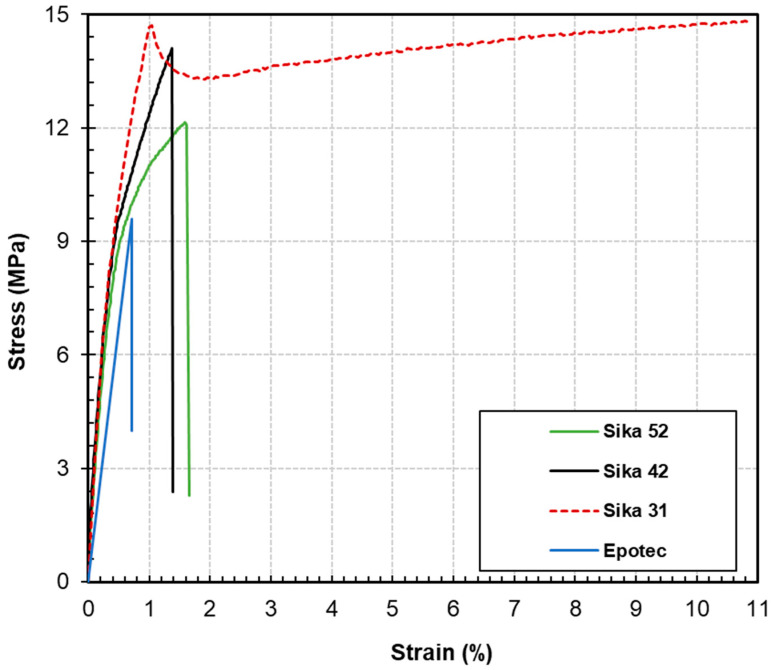
Stress-strain behavior of epoxy adhesive specimens under shear.

The typical stress-strain behavior of the epoxy adhesive specimens under shear are presented in Figure 7, and the shear properties based on average results among repeated specimens are listed in Table 6. Upon the initial application of the shear load, all adhesive samples exhibited a linear stress-strain relationship. The shear moduli of Sika 31, Sika 42, and Sika 52 were found to be very similar, ranging from 2.9 to 3.4 GPa, as illustrated in Table 6. Once the stress reached approximately 9 MPa, Epotec experienced a sudden break, leading to a shear modulus of 1.4 GPa, while the shear moduli of Sika 52 and Sika 42 gradually decreased, and their stress-strain relationships became nonlinear, with sudden breaks occurring at average stress levels of 12.1 MPa and 14.1 MPa, respectively. Notably, Sika 31 displayed a yield and strain hardening behavior similar to steel; after reaching approximately 1% strain, the stress value experienced a slight decline, followed by a rapid increase in strain as stress continued to rise. The specimen ultimately failed only after the strain exceeded 10%. Such ductile adhesive has been specially used in existing research [51,52] to fabricate ductile shear joints for FRP profiles to improve flexibility in FRP structures.

## 4. Pollutant Emission Tests

**Figure 8:**
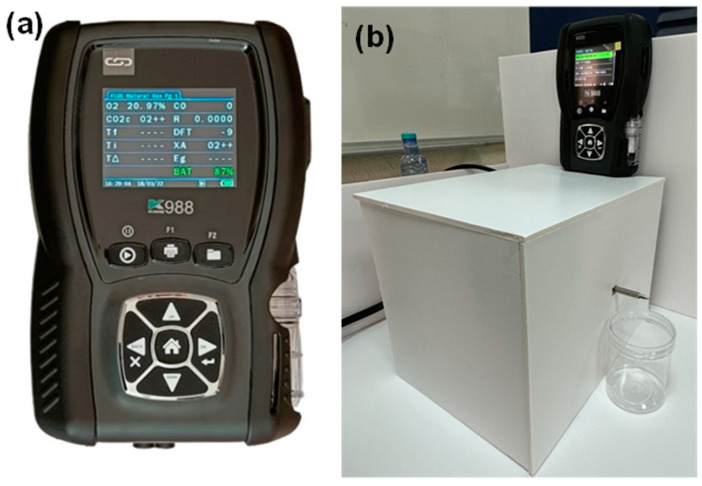
Emission tests on epoxy samples: (**a**) gas analyzer and (**b**) test setup.

In the pollutant emission tests, a Flue Gas Analyzer (KANE988) from Kane 988 Link Industrial was used as shown in Figure 8a, which is an eight-gas instrument with selectable options for CO, NO, NO_2_, NO_x_, SO_2_, and H_2_S. These sensors provide actual gas measurements rather than calculated measurements. In the experiment, 50 g samples of each adhesive were prepared. After mixing the samples, they were placed under the Flue Gas Analyzer for 120 min as shown in Figure 8b, during which the concentrations of CO, NO, NO_2_, NO_x_, and SO_2_ gases were recorded, and the results are presented in Table 7. The lab had a temperature of 25 °C and a humidity of 45–55%.

Among all of the epoxy adhesive samples, only NO_2_, NO_x_, and SO_2_ were detected, while CO and NO were not detected. Since NO_x_ represents the total concentration of NO and NO_2_, the detected levels of NO_x_ and NO_2_ are identical in the results. Therefore, Table 7 only includes the detection results for NO_2_ and SO_2_. NO_2_ and SO_2_ are critical air pollutants with significant health and environmental implications [53]. NO_2_ may pose serious health risks, particularly to the respiratory system. Short-term exposure can lead to irritation of the airways, while long-term exposure is associated with decreased lung function and increased susceptibility to respiratory infections and asthma [54]. NO_2_ plays a key role in the formation of ground-level ozone, which can lead to smog and cause harm to ecosystems [53]. SO_2_ can also cause respiratory issues. Exposure to SO_2_ has been linked to airway inflammation and the exacerbation of conditions such as asthma and chronic bronchitis. Vulnerable populations, including children and the elderly, are particularly at risk [55]. SO_2_ is a precursor to acid rain, which can severely damage forests, lakes, and soil, disrupting plant growth and aquatic ecosystems. The acidification of soil and water bodies can lead to a loss of biodiversity and altered chemical balances in ecosystems, resulting in long-term ecological harm [56].

The results in Table 7 indicate that Sika 31 only detected SO_2_, with a maximum concentration of only 2 ppm, observed at 30 to 60 min after mixing, while the other three types of adhesive samples detected both SO_2_ and NO_2_. This suggests that, from the perspective of harmful gas emissions, Sika 31 has an advantage. After 120 min of mixing, no target pollutants were detected in any of the samples. The period of higher gas concentrations was concentrated between 15 and 90 min after mixing. This indicates that the emissions of harmful gases from the four epoxy adhesives are confined to the period during application and do not have long-term effects. However, in extreme conditions like fire or high temperatures, harmful gases may be released from the cured adhesives. This still requires further investigation. During construction activities, workers should take respiratory protection measures, but there is no concern about the release of such harmful gases during their longer term use. This may be attributed to the fact that the epoxy reaction has been fully completed within 120 min, resulting in a stable solid product that is no longer volatile.

According to the Occupational Safety and Health Administration of the US, the permissible exposure limit for SO_2_ is 2 ppm, while the short-term exposure limit for SO_2_ is 5 ppm, and for NO_2_ it is 1 ppm. Therefore, the threat level of NO_2_ to health is higher than that of SO_2_. Excluding Sika 31, all other adhesives exhibited excessive levels of NO_2_ during the curing process, necessitating the implementation of protective measures.

## 5. Discussion

From the perspective of the applicable temperature, Sika 42 appears to be more suitable for the temperature and humidity conditions prevalent in Saudi Arabia, where epoxy adhesives are expected to retain their workability at elevated temperatures. Sika 31 is only effective within an applicable temperature range of 10 to 30 °C. At 40 °C, Sika 52 exhibits a pot life of merely 10 min, after which the mixture of epoxy resin and curing agent begins to harden progressively, leading to an increased viscosity that is inadequate for standard construction applications. In contrast, Sika 42 possesses a pot life of 48 min at 40 °C, rendering it more appropriate for the environment in which it was applied in this study. Furthermore, the use of Epotec adhesive requires the selection of a suitable curing agent based on the applied conditions, which can influence pot life; however, there is currently a lack of pertinent data from the manufacturer.

In terms of the aspects of the Occupational Safety and Health Administration (OSHA), Sika 42 presents significantly more potential hazards than the other epoxy adhesives examined in this research. Consequently, its application and use necessitate enhanced safety precautions. Given that Sika 42 is a three-component product, the technical requirements for its use are also comparatively higher, which places increased demands on the qualifications and training of construction personnel. In this regard, Sika 31 and Epotec are more competitive options.

Tension, compression, and shear testing reveal significant differences in the mechanical properties of the four epoxy adhesives. Sika 31 exhibits a tensile modulus of up to 10.4 GPa, whereas the moduli of the other adhesives are all below 2.7 GPa. Similarly, Sika 31 has a compressive modulus reaching 2.7 GPa, while the other adhesives do not exceed 2.2 GPa. This indicates that adherents bonded with Sika 31 experience minimal displacement under tensile and compressive loads, making it suitable for applications that require stringent limitations on tensile and compressive strain between adherents. Additionally, under shear loading, the stress-strain behavior of Sika 31 demonstrates a “yielding” behavior like steel, characterized by a rapid increase in shear strain prior to failure. Its ultimate shear strain is 10.7%, which is at least 6.6 times greater than the other samples. This behavior indicates substantial ductility, making Sika 31 appropriate for constructing ductile shear joints.

Sika 42 exhibits the highest tensile and compressive strengths among the four adhesives evaluated. Its tensile strength reaches 75.7 MPa, which is at least 1.4 times greater than that of the other adhesives. Furthermore, its compressive strength is measured at 133.8 MPa, exceeding the other adhesives by at least 80%. Consequently, Sika 42 is suitable for applications subject to substantial mechanical loads.

Sika 52 exhibited tensile and compressive moduli of 1.3 GPa and 0.8 GPa in the loading experiments, respectively, both of which were the lowest among the samples tested. This suggests that substantial deformation may occur when the structure is subjected to loads. Through appropriate design, this adhesive’s inherent deformation can be used to mitigate the impact or deformation experienced by the overall structural integration. The ultimate tensile and compressive strains were measured at 5.9% and 16.1%, respectively, both higher than those of the other adhesives. Therefore, Sika 52 is particularly suitable for applications that require the ability to accommodate significant deformation or for structural buffering.

Epotec ranks second in both tensile and compression strengths, exhibiting values of 52.9 MPa and 70.7 MPa, respectively. Consequently, it can serve as a suitable alternative to Sika 42, particularly in scenarios where the ease of application is a consideration and when subjected to significant loads. However, in shear testing, Epotec demonstrates the lowest strength, modulus, and ultimate strain, suggesting that it may lack an advantage when subjected to shear loads.

Overall, Sika 42 demonstrates superior workability under the temperature conditions prevalent in Saudi Arabia and exhibits the highest capacity to withstand tensile and compressive loads. However, as a three-component adhesive, its application is comparatively complex. Epotec ranks second in tensile and compressive stress resistance, and both Sika 31 and Epotec offer better safety and convenience relative to Sika 42. Sika 31 is particularly suitable for applications that require control of tensile strain or bonding under shear loading, given its ductility. Conversely, Sika 52 possesses the lowest tensile and compressive moduli among the four epoxy adhesives, enabling it to accommodate deformation and therefore mitigate the damages from impact loads or deformation on the overall structure.

## 6. Conclusions

To evaluate the mechanical performance, occupational health, and environmental impact of epoxy adhesives for potential applications in Saudi Arabia, this study selected four commercially available epoxy adhesives: Epotec YD 128X; Sikadur^®^-31 CF; Sikadur^®^-42 MP Slow; and Sikadur^®^-52 LP. The applicable temperature range and occupational health considerations were initially assessed based on data provided by the manufacturers. Subsequently, tensile, compressive, and shear tests were conducted to investigate the mechanical properties of these adhesives. Finally, a gas analyzer was employed to assess the major harmful gases emitted by the epoxy adhesives within 120 min following the mixing of epoxy resins and curing agents. Based on the results, the following conclusions can be drawn:

(1) Sika 42 is ideal for the temperature and humidity conditions in Saudi Arabia, with a pot life of 48 min at 40 °C. Sika 31 only operates effectively between 10 and 30 °C, while Sika 52’s pot life decreases to just 10 min under heat, underscoring Sika 42’s advantage for construction applications. Data on Epotec’s pot life vary with curing agents but are currently unavailable.
(2) Sika 42 presents greater potential hazards according to OSHA, necessitating stricter safety measures. As a three-component adhesive, it requires higher technical qualifications and a higher level of training for personnel, making Sika 31 and Epotec more accessible options.
(3) Mechanical tests show that Sika 31 has a tensile modulus of 10.4 GPa and a compressive modulus of 2.7 GPa, far exceeding other adhesives. Sika 42 boasts the highest tensile (75.7 MPa) and compressive strengths (133.8 MPa). In contrast, Sika 52 exhibits the lowest moduli, indicating significant deformation under load but effective impact absorption.
(4) Epotec ranks second for tensile (52.9 MPa) and compressive strengths (70.7 MPa), making it a viable alternative. Sika 31 excels in tensile strain control, while Sika 52 suits applications requiring flexibility against any potential impacts.
(5) Among all the epoxy adhesive samples after mixing, neither CO nor NO were detected. Only SO_2_ was detected in Sikadur^®^-31 CF, with a maximum concentration of only 2 ppm, while the other three types of adhesive samples exhibited detections of both SO_2_ and NO_2_. After 120 min of mixing, no target harmful gases were detected in any of the samples.

## Figures and Tables

**Table 1:** Applicable temperatures for different adhesives.

Product	Epotec	Sika 31		Sika 42		Sika 52	
Product use	Adhesive	Adhesive		Grouting		Sealing	
Storage temperature	N/A *	5 to 30 °C		5 to 30 °C		5 to 30 °C	
Application temperature	<80 °C	10 to 30 °C		20 to 35 °C		25 to 40 °C	
Pot life	N/A	10 °C	145 min	23 °C	105 min	23 °C	70 min
		23 °C	55 min	40 °C	48 min	30 °C	30 min
		30 °C	35 min			40 °C	10 min
Service temperature	N/A	N/A		−40 to 60 °C		N/A	

* The resin may become hazy or crystallize upon long storage, especially at low temperatures, but can be restored to its original condition by warming to 55–60 °C.

**Table 2:** Appearance and OSHA information of the epoxy adhesives.

	Epotec	Sika 31		Sika 42			Sika 52	
		Part A	Part B	Part A	Part B	Part C	Part A	Part B
Physical state	Liquid	Liquid	Paste	Liquid	Liquid	Powder	Liquid	Liquid
Color	Light yellow	Grey	Black	Clear, straw-like	Yellow	Light gray	Transparent	Yellow
Odour	Light	Epoxy-like	Characteristic	Aromatic	Amine-like	Odorless	Epoxy-like	Amine-like
Density (g/cm^3^)	1.16 (20 °C)	1.94 (20 °C)	1.95 (20 °C)	1.13 (23 °C)	0.99(23 °C)	1.85 (23 °C)	1.14 (20 °C)	0.99 (20 °C)
Viscosity (mm^2^/s)	11–14 (25 °C)	20.5 (40 °C)	N/A	>20.5 (40 °C)	>20.5 (40 °C)	N/A	>20.5 (40 °C)	7–20.5 (40 °C)
Hazard identification	H315, H319 H317, H411	H315, H319 H317, H411	H314, H412 H317	H315, H319 H317, H341	H302, H351 H314, H360 H317, H362	H335, H372 H350	H315, H319 H317, H411	H304, H318 H314, H400 H317, H411
Hazard Prevention	P261, P280 P264, P363 P272, P391 P273, P501	P261, P273 P264, P280	P261, P272 P264	P201, P264 P202, P272 P261, P280	P201, P264 P202, P263 P261, P272 P270, P280	P201, P270 P202, P271 P260, P280 P264	P261, P273 P264, P280	P273, P280
Exposure controls	Eye Hand Skin/body Respiratory	Eye Hand Skin/body	Eye Hand Skin/body Respiratory	Eye Hand Skin/body Respiratory Hygiene	Eye Hand Skin/body Respiratory Hygiene	Eye Skin/body Hygiene *	Eye Hand Skin/body	Eye Hand Skin/body
Mix ratio	N/A	A: B = 2: 1 by weight or volume		A: B: C = 5: 1: 36 by weight			A: B = 2: 1 by weight or volume	
Density (kg/m^3^)	1.16 (at 20 °C)	1.90 (at 20 °C)		2.13			1.06 (at 20 °C)	

* Specified: avoid breathing dust.

**Table 3:** Hazards Identification Codes.

Code	Specification	Code	Specification
P201	Obtain special instructions before use	P272	Contaminated work clothing must not be allowed out of the workplace
P202	Do not handle until all safety precautions have been read and understood	P273	Avoid release to the environment
P260	Do not breathe dust/fume/gas/mist/vapours/spray	P280	Wear protective gloves/eye protection/face protection
P261	Avoid breathing dust/fume/gas/mist/vapours/spray	P363	Wash contaminated clothing before reuse
P263	Avoid contact during pregnancy/while nursing	P391	Collect spillage
P264	Wash skin thoroughly after handling	P501	Dispose of contents/container in accordance with local/regional/national/international regulations
P270	Do not eat, drink or smoke when using this product		

**Table 4:** Tensile properties of tested epoxy adhesive specimens.

Epoxy Type	Tensile Strength (MPa)	Ultimate Strain	Tensile Modulus (GPa)	SD
Epotec	52.9	2.0%	2.7	1.9
Sika 31	37.5	0.3%	10.4	2.9
Sika 42	75.7	3.8%	2.0	1.1
Sika 52	48.2	5.9%	1.3	2.4

**Table 5:** Compressive properties of tested epoxy adhesive specimens.

Epoxy Type	Compressive Strength (MPa)	Ultimate Strain	Compressive Modulus (GPa)	SD
Epotec	70.7	9.4%	1.6	2.6
Sika 31	64.3	14.1%	2.7	1.5
Sika 42	133.8	9.5%	2.2	1.1
Sika 52	31.5	16.1%	0.8	1.8

**Table 6:** Shear properties of tested epoxy adhesive specimens.

Epoxy Type	Shear Strength (MPa)	Ultimate Strain	Shear Modulus (GPa)	SD
Epotec	9.6	1.2%	1.4	1.1
Sika 31	14.8	10.7%	2.9	1.9
Sika 42	14.1	1.4%	3.4	2.4
Sika 52	12.1	1.6%	3.0	1.6

**Table 7:** Emission of the adhesives after mixing (unit: ppm).

Time	Epotec		Sika 31		Sika 42		Sika 52	
	NO_2_	SO_2_	NO_2_	SO_2_	NO_2_	SO_2_	NO_2_	SO_2_
5 min	0	1	0	0	0	0	0	0
10 min	0	1	0	0	0	0	2	2
15 min	2	3	0	1	2	2	3	3
30 min	3	4	0	2	2	3	3	3
60 min	2	3	0	2	3	5	4	4
90 min	0	1	0	0	0	0	5	5
120 min	0	0	0	0	0	0	0	0

## Data Availability

The datasets used during the current study are available from the corresponding author upon reasonable request (due to privacy of ongoing research project).
